# Case Report: The Rehabilitation of a Patient with Acute Transverse Myelitis after COVID-19 Vaccination

**DOI:** 10.3390/clinpract14030085

**Published:** 2024-06-06

**Authors:** Piotr Niebrzydowski, Małgorzata Kusiak-Kaczmarek, Jarosław Tomaszewski, Maciej Gmiński, Dominika Szalewska

**Affiliations:** 1 Rehabilitation Clinic, University Clinical Center, Aleja Zwycięstwa 30 Street, 80-219 Gdańsk, Poland; mkkaczmarek@gumed.edu.pl (M.K.-K.); mgminski@uck.gda.pl (M.G.); 2 Division of Rehabilitation Medicine, Faculty of Health Sciences, Medical University of Gdansk, 80-219 Gdańsk, Poland; jtomaszewski@gumed.edu.pl

**Keywords:** COVID-19 vaccine, transverse myelitis

## Abstract

We report the case of a 55-year-old man with multi-symptomatic transverse myelitis after vaccination against coronavirus disease 2019 (COVID-19). The patient was diagnosed based on the course of the disease and the results of imaging and laboratory tests. We excluded other most probable causes of the disease. The quick start of diagnosis allowed for early treatment with intravenous steroids and then plasmapheresis and the implementation of modern rehabilitation methods using biofeedback platforms, among others, and an exoskeleton. The patient returned to work, but the rehabilitation process continues to this day due to persistent symptoms that impair the patient’s quality of life.

## 1. Introduction

Thanks to the vaccine, the spread of the COVID-19 virus was stopped, and many lives were saved—especially those of individuals with underlying health conditions. However, the vaccine was only recently developed, and its side effects, especially its late side effects, are still being discovered.

The most common side effects of the COVID-19 vaccine resemble the side effects of other popular vaccines. Nevertheless, serious side effects can also occur. These include thrombosis, thrombocytopenia, myocarditis, pericarditis, and anaphylaxis, as well as neurological side effects [1], such as Guillain–Barré syndrome, venous sinus thrombosis, facial nerve palsy, small fiber neuropathy, and multiple sclerosis, which rarely occur [2]. For example, 593 case reports of transverse myelitis were found in the scientific literature encompassing 11.7 billion doses of the vaccine, according to Raethke et al. [3].

We report the case of a patient who developed transverse myelitis after being vaccinated against COVID-19. He was treated with, among other treatments, plasmapheresis in the neurology department. Then, he was directly referred to the rehabilitation clinic, which he left after two months. After completing all the inpatient rehabilitation courses, he was able to return to work.

Transverse myelitis is a neurological disorder. Depending on the etiology, 1–4 new cases per 1 million people are observed annually. It affects men and women equally, occurring most frequently in people aged 20–40 years [4]. Transverse myelitis can develop due to various causes, such as viral and bacterial infections, spinal cord infarction, systemic lupus erythematosus, Sjörgen’s syndrome, systemic sclerosis, antiphospholipid antibody syndrome, ankylosing spondylitis, rheumatoid arthritis, intoxications, or multiple sclerosis. It may also occur idiopathically as part of paraneoplastic syndrome or as a side effect of vaccination [5].

## 2. Case Presentation

On 3 October 2022, a 175 cm tall, 55-year-old man weighing 91 kg, who is a professionally active veterinarian with chronic hypertension and gout, was admitted to the emergency department (ED) at the University Clinical Centre (UCC) in Gdańsk with the following acute symptoms: tightness in the chest, burning pain radiating from the chest to both upper extremities, and sensory impairment at the nipple level (T4). After several minutes, bilateral arm paresis appeared. The patient admitted that he had been given a fourth dose of the Pfizer COVID-19 vaccine three weeks prior. ED admission excluded cardiological and vascular causes. A few hours later, the strength of his lower left extremity deteriorated (from 5 to 2 on Lovett’s scale) [6,7].

**Figure 1:**
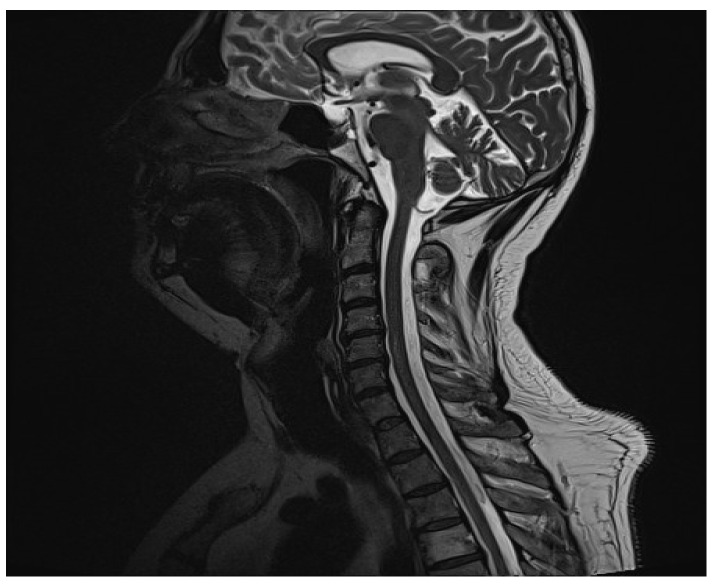
The MRI of the cervical spine on the T2-weighted short tau inversion recovery scan showed a hyperintense lesion on C5–C6.

During the patient’s hospital stay, a constant weakening of the lower extremities (the right going from 5 to 3 on Lovett’s scale, the left going from 2 to 0 on Lovett’s scale) [6,7] followed by a weakening of the upper extremities (both going from 5 to 3 on Lovett’s scale) [6,7] was observed. On MR images, a radiologist found an inflammatory focus in the central part of the C5/C6 spinal cord (Figure 1). The use of contrast agents did not reveal any activity features. The patient was transferred to the neurology clinic, where more diagnostic tests were performed. The MR images of the cerebrum and the thoracic spine acquired later showed no lesions. CSF test results were typical. The biochemical CSF examination revealed elevated total protein (0.91 g/L) and elevated albumin (656.7 mg/L) levels. Intradural type 4 oligoclonal band synthesis, as defined in the Charcot Foundation standards, was not observed. The CSF culture test and PCR test for the presence of pathogens in CSF were negative. The fluid culture test was negative. The aquaporin-4 antibody test was negative. The result of the anti-MOG antibody test performed on 13 October 2022 was slightly elevated (1:10). The result of another trial, conducted on 18 October 2022, was <1:10, which is described as unfavorable. Then, connective tissue disorders were ruled out, and the patient was referred for a rheumatological consultation.

Initial treatment consisted of the administration of 1 g of methylprednisolone intravenously once per day. Seven doses were given. After that, due to the patient’s unsatisfactory response to the treatment, the patient underwent five plasmapheresis sessions. The patient was consulted by a rehabilitation specialist.

On October 13, the day of the consultation, the patient was able to move in a wheelchair, eat independently, and brush his teeth. He experienced weakened strength in his upper limbs (4 on the Lovett’s scale), lower right limb (3 on the Lovett’s scale), and lower left limb (0 on the Lovett’s scale) [6,7].The patient was catheterized and was unable to urinate without the catheter (Table 1).

The patient was treated for mobility issues and was found eligible to be transferred to the rehabilitation clinic at the UCC. 

**Figure 2:**
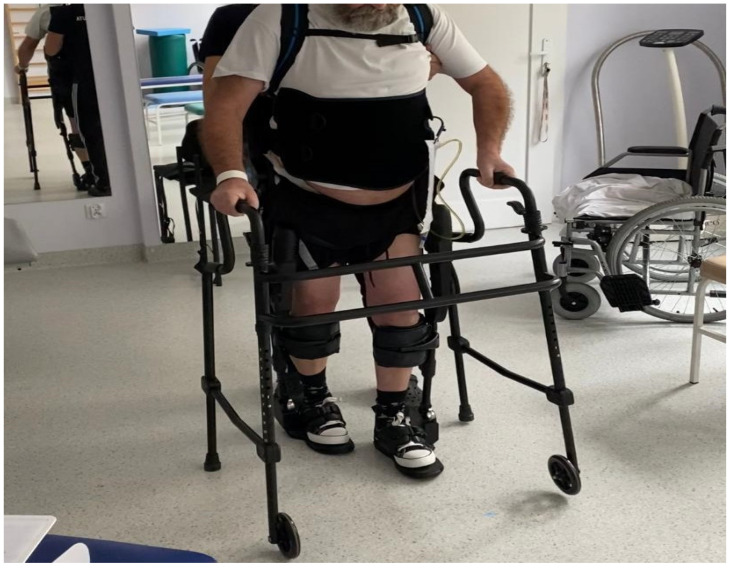
The patient rehabilitated with an exoskeleton.

Treatment resulted in a significant improvement in the function of the upper extremities and a partial revision of the role of the lower extremities. On 18 October 2022, the patient was transferred to the rehabilitation clinic. At the moment of admission, the patient was undergoing verticalization, required help with self-care, and had no sphincter control. The patient also suffered from significant lower left extremity paresis and paresthesia, as well as bilateral sensory impairment below T8. The patient underwent rehabilitation for approximately two months. This consisted of general rehabilitation exercises, isometric core muscle exercises, verticalization and gait training, PNF exercises, biofeedback exercises, exoskeleton exercises (Figure 2), and the electrical stimulation of the left gluteal muscles. Kinesitherapy in the rehabilitation clinic took place four hours a day, six days a week, from the first day of hospitalization. Biofeedback exercises were added on November 24. During these exercises, the patient was able to stand on the interactive platforms and, among other things, practice balancing for approximately half an hour every day.

An exoskeleton training program was conducted three times per week, with each session lasting 60–90 min (total number of sessions: 16). Each training session included walking on a flat indoor surface. No falls or injures, such as fractures, occurred. Regular assessments of the patient’s mental health performed as part of hospital care showed that it was good. At the end of the hospital stay, the patient spoke about his plans for the future and the subsequent rehabilitation course.

**Figure 3:**
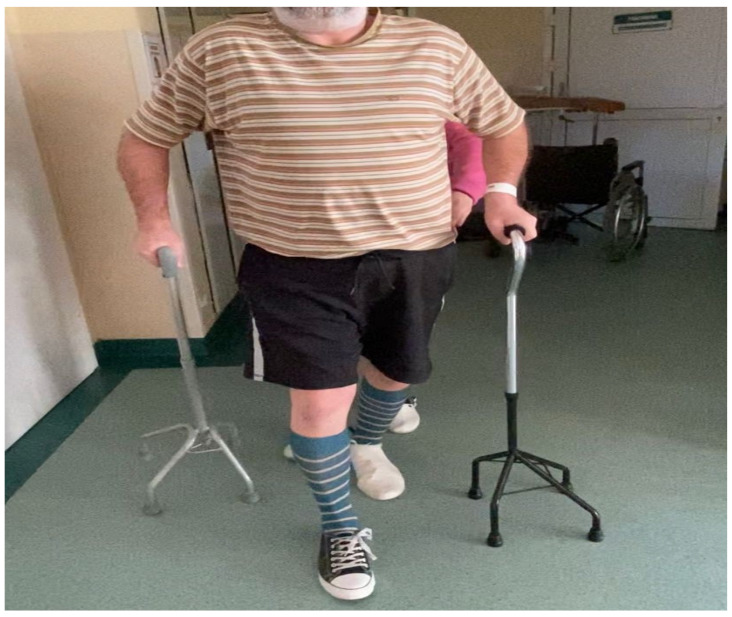
The patient used two quadruped canes for walking at the end of rehabilitation in the clinic.

When the hospital rehabilitation of the patient finished, the lower left extremity paresis subsided to a degree that allowed him to move on his own using two elbow crutches or two quadrupted canes (Figure 3), including moving on flights of stairs. To cover longer distances, the patient used a wheelchair. Moreover, sensory impairment subsided, and the patient regained sphincter control. The patient had his urinary catheter removed on 17 November, 1.5 months after the onset of the disease. He has not experienced urinary stasis or incontinence since then. After bowel movement improved, lactulose was discontinued (Table 1). The patient became less dependent on assistance.

The patient had the following scores on admission: Rankin scale: 5, Barthel scale: 6. The patient had the following scores on discharge: Rankin scale: 2, Barthel scale: 17. The patient spent 2 months in the rehabilitation ward.

The patient left the clinic on 20 December 2022. He was able to move up and down stairs with the help of elbow crutches. Although he was unable to bathe himself, he was able to use the toilet and dress himself. Immediately after leaving the clinic, the patient underwent further rehabilitation at a provincial hospital in Jelenia Góra. During a recent phone call (28 August 2023), the patient claimed to be able to move around using a walking stick. He also mentioned using a brace for his dropping foot on the left side (Table 1). The patient takes Nivalin. He returned to work as a vet and is still supervised by a rehabilitation specialist and a neurologist.

## 3. Discussion

Transverse myelitis can occur at any level of the spine. However, it affects the thoracic region most frequently [4]. Symptoms include pain in the extremities, torso girdle pain, paresthesia, paresis in the extremities, and sphincter impairment. They worsen within a period ranging from a few hours to a few weeks [5,8]. 

Diagnosis should include in-depth history-taking, a physical examination, an MRI of the spinal cord utilizing a gadolinium-based contrast agent within four hours of onset, a laboratory blood test, a laboratory CSF test, and ruling out brain demyelination based on an MRI utilizing a contrast agent containing gadolinium.

In 2002, the Transverse Myelitis Consortium Group proposed diagnostic criteria for transverse myelitis [9]. These criteria were met in the case being discussed: the patient showed bilateral symptoms that gradually became more pronounced and corresponded with the level of inflammation according to the MRI images. Moreover, the disorder progressed to the nadir in less than 21 days following onset. Apart from that, multiple sclerosis; neuromyelitis optica; lesions in other parts of the spinal cord and in the brain; and infectious, vascular, and neurological diseases were excluded based on diagnostic tests. Laboratory tests excluded the following infections: varicella zoster, cytomegalovirus, herpes simplex virus, influenza A and B, HIV, human herpesvirus 6, SARS-CoV-2, enterovirus, toxoplasmosis, human parechovirus, Haemophilus influenzae, Listeria monocytogenes, Treponema pallidum, Neisseria meningitidis, Streptococcus A and B, Borrelia burgdorferi, Mycobacterium tuberculosis, and Cryptococcus neoformans. Other infectious causes of transverse myelitis include hepatitis A, hepatitis B, hepatitis C, hepatitis E, measles, mumps, rubella, varicella zoster, Epstein–Barr, cytomegalovirus, herpes simplex, influenza A/B, lymphocytic choriomeningitis virus, chikungunya, hantavirus, HIV, human T-cell lymphotropic virus, human herpes virus 6, Japanese encephalitis, Murray Valley encephalitis, St. Louis encephalitis, tick-borne encephalitis, vaccinia, Rocky Mountain spotted fever, dengue virus, enterovirus 71, coxsackievirus A and B, West Nile virus, parvovirus B19, echovirus, Mycoplasma pneumoniae, Campylobacter jejuni, Acinetobacter baumanii, Coxiella burnetii, Bartonella henselae, Chlamydia psittaci, Leptospira, Chlamydia pneumoniae, Legionella pneumonia, Orientia tsutsugamushi (scrub typhus), Salmonella paratyphi B, and Brucellosis melitensis. Differential diagnosis can be extended for fungal and parasitic infections by performing tests for Actinomyces, Blastomyces, Coccidioides, Aspergillus, Cladophialophora bantiana, and Toxocara species and for Schistosoma species, Gnasthostoma spinigerum, Echinococcus granulosus, Taenia solium, Acanthamoeba species, Paragonimus westermani, and Trypanosoma brucei. To exclude paraneoplastic etiology, the following tests should be performed: anti-Ri (ANNA-2) antibody, CRMP-5-IgG antibody, anti-amphiphysin IgG antibody, anti-GAD65 antibody, and NMDAR antibody tests [4].

Transverse myelitis can also develop in the course of myelin oligodendrocyte antibody-associated disease (MOGAD) and occurs in about 25% of MOGAD cases. The diagnosis of MOGAD should be performed using an MOG antibody titer >1:100. Higher dilution will result in a higher number of false positives, which could lead to incorrect diagnosis and treatment. The administration of steroids or immunoglobulins prior to plasmapheresis may increase the risk of false-negative results. If the patient receives such treatment, the test should be repeated at least three months after the end of that treatment. In patients with multiple sclerosis, the results of an MOG antibody titer may be falsely positive [10].

After excluding the most probable causes of transverse myelitis, the authors began to suspect that this case could be associated with the fourth dose of the Pfizer COVID-19 vaccine taken by the patient shortly before the onset of symptoms. Many cases of transverse myelitis after COVID-19 vaccination were reported in 2021, which the case being discussed resembles [11]. No pathogen was found in the CSF sample taken from the patient. The course of treatment was also similar to that in the aforementioned cases. However, the rehabilitation process cannot be compared, as it was not described in the cases mentioned above. Elevated protein levels in the CSF and negative results of serology tests are consistent with the results of the majority of patients with transverse myelitis that occurred after either COVID-19 vaccination or infection [12]. 

The SARS-CoV-2 spike protein antibody directly reacts with myelin. Additionally, the interaction of the spike protein with ACE2 receptors present in neurons results in a demyelination process [13].

In addition, alpha7 nAChR, another host receptor of the SARS-CoV-2 spike protein [14], plays an important role in stopping the development of the disease. alpha7 nAChR agonists, such as galantamine, the active ingredient of Nivalin, which the patient is currently taking, activate the anti-cholinergic anti-inflammatory pathway.

This pathway can also be activated via non-invasive Vagus Nerve Stimulation (nVNS) [15], which could turn out to be a true game changer not only in etiologic rehabilitation [16] but also in symptomatic rehabilitation, notably acting on motor rehabilitation [17] and pain [18]. 

Treatment should focus on treating the cause of the transverse myelitis onset, which involves the administration of antivirals, corticosteroids, or cyclophosphamide. Other modalities that may be utilized are plasmapheresis, immunoglobulin infusion [4], rehabilitation, and symptomatic treatment, such as the use of baclofen or gabapentin in cases of spasticity or physiotherapy and pharmacotherapy in cases of pain [19]. Currently, the patient takes Nivalin. The active ingredient of Nivalin is galantamine, which is used for the treatment of Alzheimer’s disease. Animal testing shows that galantamine increases neural tissue survival and accelerates motor function recovery following acute spinal cord injury [20]. 

At the neurology department, the patient presented multiple symptoms of acute transverse myelitis. The treatment involved corticosteroid administration and plasmapheresis. One article [21] concluded that, in patients with multiple motor-sensory symptoms and sphincter symptoms, the prognosis regarding the reversal of symptoms is poor. Other researchers described a case of acute transverse myelitis that occurred after a COVID-19 infection similar to the one being discussed herein, and proposed to treat such patients using steroids and plasmapheresis or immunoglobulins in cases involving elevated IL-6 levels [22]. 

The symptoms of transverse myelitis include problems passing urine and stools. Spinal cord damage can cause urinary incontinence and urinary retention, as well as neurogenic bowel dysfunction [19], which includes symptoms such as excessive anal sphincter activity and weak bowel movements. The patient in the current case showed these symptoms. At the neurology department, the patient was given lactulose due to difficulty passing stools. The patient also had a catheter, since he lost sphincter control. These disorders subsided before the end of the patient’s hospitalization. The improvement in the patient’s state can be attributed to the fact that the patient followed a diet made with his symptoms in mind, as well as verticalization and gait retraining, which improved bowel movement. In many patients suffering from transverse myelitis, the aforementioned impairments do not subside, necessitating further urological rehabilitation, the administration of anticholinergic agents, and intermittent catheterization, which the patient encounters during their hospital stay. It is possible that to improve the function of an overactive bladder without the use of drugs and intermittent catheterization in the future, patients will be able to use electrical stimulation of the genital nerves (GNS) on a permanent basis at home instead. A small sample study showed that GNS reduced urinary incontinence symptoms and, according to the study participants, improved participants’ quality of life. The participants expressed interest in using an implanted GNS system. According to the study participants, the system used in the study was too large, and the length of its electrodes was not suitable [23].

During rehabilitation, the patient underwent electrical stimulation of the left gluteal muscles to help maintain strength and decrease muscle atrophy [19]. As part of neurorehabilitation, the patient performed exercises using an exoskeleton. Apart from improving bowel movements, the use of an exoskeleton during rehabilitation decreases spasticity, reduces back pain, improves the mental state of the patient, and gives the patient motivation to continue rehabilitation [24,25].

The patient increased his number of steps and improved his gait quality each session. The patient tolerated each training well and noticed improvements in each week of rehabilitation. During the entire four-month rehabilitation period, the patient did not report any problems with spasticity and reported improvements in bowel movement regulation, which could be related to consistent exoskeleton training [24].

Exoskeleton use is proof that brain–computer interface technology is advancing and becoming more available. The brain–computer interface is a system that translates central nervous system signals into command signals for an external or internal device. Further development of this technology may help patients suffering from neurological diseases, such as transverse myelitis, and improve motor function, mobility, bowel movement, bladder function, sexual function, and many more areas by bypassing the damaged part of the nervous system or reorganizing the nervous system to regain organ function. Another emerging role of brain–computer interfaces is improving diagnostic precision in disorders of consciousness. Currently, many human and animal studies are being conducted to improve brain–computer interface technology. Measures should be taken to improve the availability of such solutions in more healthcare facilities in the future and to make them less expensive and more reliable. Additionally, more attention should be devoted to teaching medical personnel how to use emerging technologies [26].

## 4. Conclusions

Transverse myelitis occurring as an adverse event following immunization is not unprecedented. The use of an exoskeleton 28 days after the onset of the disease facilitated early gait re-education and the prevention of muscle atrophy. Utilizing new technologies will make it possible to improve the quality of life of patients whose symptoms do not subside after treatment and rehabilitation. The patient returned to work after a quick, correct diagnosis, adapted treatment, and four months of rehabilitation after spinal cord inflammation.

## Figures and Tables

**Table 1:** The assessment of the patient’s condition before and after rehabilitation.

Prior to Rehabilitation	Following Rehabilitation
Patient moved in a wheelchair by himself.	Patient moves using a walking stick and uses a brace for his dropping foot on the left side.
Patient demonstrated weakened strength in his upper limbs (4 on the Lovett’s scale).	Patient demonstrated proper strength in his upper limbs (5 on the Lovett’s scale).
Patient demonstrated weakened strength in lower right limb (3 on the Lovett’s scale).	Patient demonstrated proper strength in his lower right limb (5 on the Lovett’s scale).
Patient had paralysis of the lower left limb.	Patient has a dropping foot on the left side.
Patient had urinary stasis and bowel incontinence.	Patient regained sphincter control.

## Data Availability

It can be obtained from the article.
